# Can the tardigrade *Hypsibius dujardini* survive in the absence of the geomagnetic field?

**DOI:** 10.1371/journal.pone.0183380

**Published:** 2017-09-08

**Authors:** Weronika Erdmann, Bogdan Idzikowski, Wojciech Kowalski, Bogdan Szymański, Jakub Z. Kosicki, Łukasz Kaczmarek

**Affiliations:** 1 Department of Animal Taxonomy and Ecology, Faculty of Biology, Adam Mickiewicz University, Poznań, Umultowska 89, Poznań, Poland; 2 Institute of Molecular Physics, Polish Academy of Sciences, M. Smoluchowskiego17, Poznań, Poland; 3 Department of Avian Biology and Ecology, Faculty of Biology, Adam Mickiewicz University, Poznań, Umultowska 89, Poznań, Poland; Medical University Graz, AUSTRIA

## Abstract

Earth's geomagnetic field has undergone critical changes in the past. Studies on the influence of the magnetic field on Earth’s organisms are crucial for the understanding of evolution of life on Earth and astrobiological considerations. Numerous studies conducted both on plants and animals confirmed the significant influence of the geomagnetic field on the metabolism of living organisms. Water bears (Tardigrada), which are a mong the most resistant animals due to their cryptobiotic abilities, show significant resistance to a number of environmental stressors, but the influence of the geomagnetic field on their fitness has not been addressed before. In our studies, we used eutardigrade *Hypsibius dujardini* to analyse whether isolation from the geomagnetic field had an effect on mortality. We found that *Hypsibius dujardini* specimens demonstrated relatively high mortality during anhydrobiosis, also in control groups exposed to the normal geomagnetic field. Moreover, similar mortality was observed in anhydrobiotic specimens isolated from the geomagnetic field. However, a significant difference was noted between tardigrade survival and the moment of their isolation from the geomagnetic field. In particular, tardigrade mortality substantially increased in absence of a magnetic field during the process of entering anhydrobiosis and returning to active life. Our results suggest that these processes rely on complex metabolic processes that are critically influenced by the geomagnetic field.

## Introduction

A geomagnetic field is a vector field which means that its intensity and direction at a given place fully characterise it. Earth’s magnetic poles are currently located near its geographical poles [1]. It is highly possible that in its early days the Earth did not have a magnetic field. However, it is a well-known fact that a geomagnetic field existed when life appeared on the planet [1] approx. 3.5 billion years ago [2, 3], and it is maintained by the so-called self-inducting dynamo [4].

The geomagnetic field influences life on Earth in many ways. Undoubtedly, any weakening of the geomagnetic field or total isolation from it causes interference with a number of metabolic processes. Numerous studies have supported a hypothesis that the geomagnetic field has a significant effect on the metabolism of living organisms, mostly on bacterial cells, animal tissue and internal organs [5], as well as plants [6, 7, 8], but to a lesser degree on animals, *sensu stricto* [9, 10].

Any changes in the magnetic field may influence biochemical reactions, e.g. through an increase or decrease in enzyme activity. For example, the activity of hydroxindolo-O-methyltransferase (HIOMT), an enzyme responsible for melatonin biosynthesis in the pineal gland and retina, and of serotonin N-acetyltransferase (NAT), is altered when changes in the magnetic field appear [11]. Weak constant magnetic fields (i.e. the geomagnetic field) and weak variable magnetic fields influence the transport of ions and impair the function of certain enzymes [12, 13]. For instance, the redox activity of cytochrome c oxidase is modulated both by static and slightly alternating, weak and moderate magnetic fields [12]. It is also highly possible that the magnetic field can influence the activity of calmodulin [13, 14]. Changes in magnetic fields *via* changes to chromatin conformation and transcription stimulation may have a direct effect on DNA molecules [15]. By increasing or decreasing enzyme activity, the effect of magnetic fields may also lead to a greater frequency of cell division, and in consequence to an organism’s growth [16]. Therefore, by affecting biochemical reactions, magnetic fields may influence the physiology of the entire organism.

Individual research conducted by the National Aeronautics and Space Administration (NASA) on mice has shown that long-term absence of a magnetic field significantly reduces the fitness of the tested animals [9]. It has been also observed that not only do hypomagnetic conditions cause changes in animal behaviour, e.g. loss of activity, but also induce significant histological changes in tissue, lead to hair loss, and an increase in mortality [9]. Studies on rats have demonstrated that shielded from the geomagnetic field, their hair suffers from reduced levels of certain elements, including Fe, Mn, Cu, and Cr [10]. In humans, long periods of absence of the magnetic field induce circadian rhythm disorder, a decrease in metabolism, gastrointestinal diseases, and a decrease in the number of leukocytes [5]. Unfortunately, experiments with the impact of absent or weak geomagnetic fields on animals have been rare and were generally limited to vertebrates. Invertebrates, especially cryptobiotic taxa, have been completely ignored in such experiments.

Different groups of extremophiles can be expected to have the greatest chance to survive in hypomagnetic conditions thanks to their high resistance to different environmental stressors. The groups include some bacteria and archaea, many species of rotifers and nematodes, some arthropods (mainly shellfish), and tardigrades [17, 18].

Tardigrades (water bears), one of the toughest metazoans on Earth and model multicellular organisms, are often used in studies on survivability in extreme conditions [19, 20]. A number of experiments proved that some tardigrade species displayed significant resistance to a number of environmental stressors, including desiccation [21], very high and sub-zero temperatures [22, 23, 24], extreme levels of ionizing radiation [25, 26], high pressure [23, 27, 28], as well as chemicals, such as ethanol [23], carbon dioxide, hydrogen sulphide [19], 1-hexanol [23, 29], and methyl bromide gas [30]. They are also able to survive in the vacuum of space, even in combined exposure to a space vacuum, and solar and cosmic radiation [31]. Many species of tardigrades owe this remarkable resistance to adverse conditions to their ability to enter cryptobiosis. During this state, metabolic processes significantly decrease or even stop [32, 33, 34]. But entering cryptobiosis and returning to active life requires preparation in the form of specific metabolic processes. These preparations include, for example, forming a tun [29] and synthesising many different bioprotectants. Possible bioprotectants include non-reducing sugars, such as trehalose [35] or other molecules, e.g. LEA (late embryogenesis abundant), HSP (heat shock proteins), CAHS (cytoplasmic abundant heat soluble), SAHS (secretory abundant heat soluble), and aquaporin proteins [19, 21, 36, 37, 38, 39]. The main role of these molecules probably is to protect the cell’s macromolecules and other structures from the consequences of desiccation/dehydration. Despite all these adaptations, many environmental factors can still cause serious damage to some macromolecules, including DNA strands [40], even though the tardigrades may have developed very efficient DNA repair mechanisms [21, 41]. These negative environmental factors can include the absence of a magnetic field, although the influence of changes in magnetic fields on tardigrade metabolic or biochemical activity has never been tested. However, as stated in the previous paragraphs, the phenomenon is quite well documented in the case of other groups of organisms. When extrapolating research results to tardigrades, we had considered whether the mechanisms and adaptations used by tardigrades to prepare for the cryptobiotic state and the return to active life could be affected when isolated from the geomagnetic field.

Until now, all experiments on the resistance of tardigrades to different stress conditions had been conducted under the cover of the geomagnetic field. This study is the first to test the impact of isolation from the geomagnetic field on the mortality of the *Hypsibius dujardini* (Doyère, 1840) at different life stages, i.e. in the active state, upon entering cryptobiosis, during cryptobiosis, and when returning to active life.

The aim of the study is as follows: **a)** to examine the impact of isolation from the magnetic field on the survival of the *H*. *dujardini* survival, and **b)** to investigate differences between *H*. *dujardini* mortality and the moment of isolation from the geomagnetic field.

## Materials and methods

### Animal model

The studies were carried out on the eutardigrade *Hypsibius dujardini*, which is an amphibious and parthenogenetic species that feeds on algae. This species is relatively easy to culture and it is recognised as a perfect model species to study tardigrade evolution and development, as well as UV and gamma radiation resistance, genome structure, and anhydrobiosis [42, 43, 44, 45, 46, 47, 48].

The initial specimens in our culture were obtained from a culture, which was cultivated at the Department of Entomology, Institute of Zoology at the Jagiellonian University and provided by Łukasz Michalczyk, PhD. The specimens in these cultures came from rotting leaves collected in 1987 by Robert McNuff from a pond in Darcy Lever (Sciento Company, United Kingdom, Greater Manchester, Bolton; 53°33'32''N, 02°23'48''W; 75m asl). The tardigrades were cultured in 250 ml transparent plastic bottles in 130 ml of medium, consisting of. distilled water with a mixture of two species of algae, *Chlorella* sp. and *Chlorococcum* sp. (Sciento Company, JA68) added *ad libitum* as a source of food. The medium was replaced every two months. The cultures were constantly monitored in order to control the tardigrade density. When the level of density was very high (more than approx. 2,000 specimens/bottle), we separated cultures into new containers to avoid overcrowding and increased mortality.

### Anti-magnetic chamber (CIMF—Chamber isolated from magnetic field) (Fig 1)

There are four major methods applied to reduce the ambient magnetic field: 1) superimposition of fields; 2) astatisation; 3) shielding; and 4) compensation, from which we chose shielding for our experiments. According to a NASA technical note, “Shielding of the subject from the geomagnetic field can be accomplished by surrounding the experimental region completely with metal sheets of very high magnetic permeability (…)” [9]. All the experiments were carried out inside a special magnetic field shielding chamber of our own design, which had the form of a double cylinder closed at both ends with a double-layered lid. The chamber was made from 1 mm thick μ-metal, which was a soft magnetic alloy composed mainly of nickel and iron (approximately 77% nickel, 16% iron, 5% copper and 2% molybdenum). It was able to deflect the force field by concentrating it inside the material’s substance [9], and it ensured a 200-fold reduction of the geomagnetic field [49, 50].

**Fig 1:**
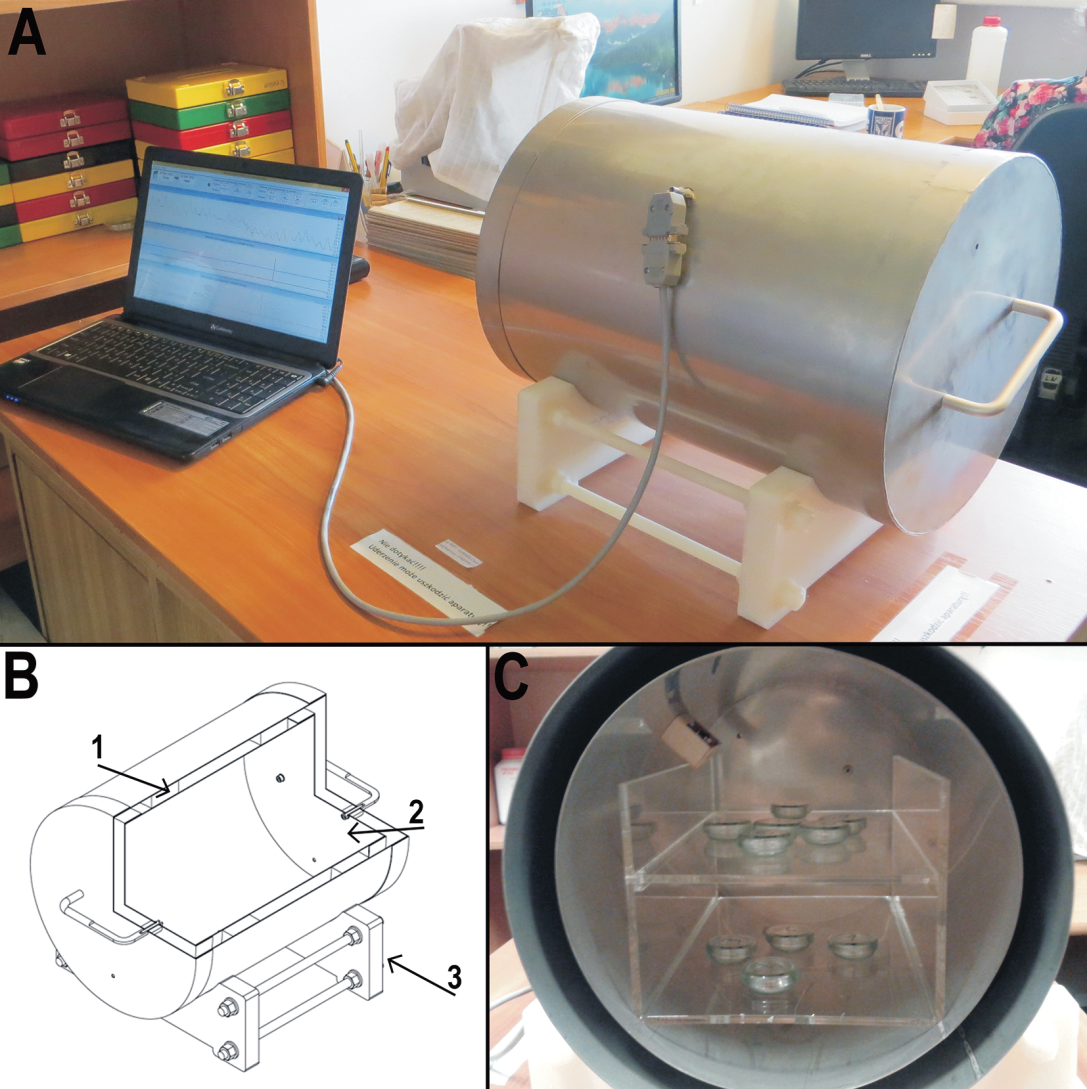
Anti-magnetic chamber (CIMF—Chamber isolated from magnetic field). A—chamber during work, connected with laptop, B—schematic graph of the chamber (1–1 mm thickness mu-metal composed of nickel and iron, 2—inside of the chamber, 3—plastic base), C–inside of the chamber, shelves with tardigrade cultures in Petri dishes.

To control and compare the geomagnetic field inside and outside the shield chamber, we used extremely sensitive three-axis type KMZ10A magnetic field sensors, made by Royal Philips Electronics. These sensors employed the magnetoresistive effect of the permalloy thin-film. Signals from each sensor were converted from analogue to digital 16-bit two channel AD7705 converters that were connected to a micro-controller. Additionally, an Atmega8 micro-controller computed digital signals from a 12-bit DS18B20 sensor placed within the shield chamber. All measured parameters, such as the strength of the magnetic field outside and inside the chamber (measured in μT), and air temperature inside CIMF (measured in °C) were transmitted through a USB interface to the computer, then collected and presented on the computer screen.

### Control box

The Control Box was an opaque plastic container (18.5x12x33 cm), uninsulated from the magnetic field. The Control Box was used for control groups to provide conditions (isolation from light and limited air movement) similar to those inside the chamber, but without isolation from the magnetic field, during the course of the experiments.

### Experiments

We conducted three experiments: a) with specimens in anhydrobiosis, b) with specimens entering anhydrobiosis, and c) with specimens returning to active life. In every experiment, 600 specimens (300 in the control group and 300 in the test group) of *H*. *dujardini* were used. In each experiment, 10 replicates were performed (30 specimens in the control and 30 in the test group). As we were unable to determine the age of each specimen in a culture, all specimens used in the experiments were selected randomly and were of different ages. Following the culture procedure, individual specimens were no younger than 5 days and no older than 15. During the dehydration process, we were unable to control humidity using electronic sensors and/or hygrometers, because solenoid is an integral part. Due to this fact, the active devices generate a magnetic field which we would not be able to control and which would affect experiment results. Therefore, we had to use a different method to estimate relative humidity in the chamber.

In each experiment, the process of dehydration lasted three days. Specimens were kept in closed Petri dishes (⌀ = 40 mm) in 300 μl of distilled water. The volume was calculated according to the following equation [51, 52]:

$$M_{W_{add}}=\left(\frac{M_{a}\times \frac{\frac{\phi_{99}\times p_{s}}{100}\times m_{w}}{\frac{\phi_{99}\times p_{s}}{100}\times m_{w}+(P_{b}-\frac{\phi_{99}\times p_{s}}{100})m_{a}}}{1-\frac{\frac{\phi_{99}\times p_{s}}{100}\times m_{w}}{\frac{\phi_{99}\times p_{s}}{100}\times m_{w}+(P_{b}-\frac{\phi_{99}\times p_{s}}{100})m_{a}}}\right)-\left(\frac{M_{a}\times \frac{\frac{\phi_{30}\times p_{s}}{100}\times m_{w}}{\frac{\phi_{30}\times p_{s}}{100}\times m_{w}+\left(P_{b}-\frac{\phi_{30}\times p_{s}}{100}\right)m_{a}}}{1--\frac{\frac{\phi_{30}\times p_{s}}{100}\times m_{w}}{\frac{\phi_{30}\times p_{s}}{100}\times m_{w}+\left(P_{b}-\frac{\phi_{30}\times p_{s}}{100}\right)m_{a}}}\right)$$

where:

*M*_*wadd*_*−*mass of water which we had to add
*M*_*w99*_ –mass of water in the air with 99% relative humidity
*M*_*w30*_ –mass of water in the air with 30% relative humidity
*M*_*a*_−mass of dry air = ma x (Pb–p) V / RT (41093,332·10–6 g)
*m*_*w*_−molecular mass of water (18.016 g/mol)
*m*_*a*_−molecular mass of air (28.966 g/mol)
*p*_*s*_*−*saturation pressure of water at the temperature of the ambient
*P*_*b*_−total or barometric pressure (average air pressure during the experiments was 100900 Pa)
*ϕ* –relative humidity expressed as a percentage according to David R. Lide (2005) is defined as the ratio of partial pressure of water vapour in the air (p) to the saturation of vapour pressure (ps) of water in the same temperature [51] and expressed by equation (2) [52].

$$\phi =100\frac{p}{p_{s}}$$

where:

*p*–partial pressure of water vapour present in the ambient air
*p*_*s*_−saturation pressure of water in ambient temperature (2062.83 Pa)

In our experiment, after the water in tightly sealed (with parafilm) Petri dishes (⌀ = 40mm) had evaporated at a temperature of 18°C, we obtained a humidity reading of approx. 99.0%, and in such conditions, dehydration of the tardigrades lasted two days. After preconditioning, the Petri dishes were opened for one day in a laboratory room in a average temperature of 23.9°C and humidity of 30% to complete the dehydration procedure. Such a procedure is necessary for species like *H*. *dujardini*, because of their low tolerance to desiccation, to be able to enter anhydrobiosis successfully [47, 53]. In addition, they require longer dehydration at high humidity levels before they can survive exposure to much lower humidity and total desiccation. Test groups were kept either in the isolating chamber or, when necessary, in a box that was identical to the Control Box. Control groups were kept in the Control Box. Both the control and test groups of tardigrades were placed in Petri dishes (ø = 40mm). All experiments were conducted for 21 days in the same laboratory room in a temperature of 23.9°C.

In all experiments, specimens were considered dead if they did not display visible signs of life (movements of the body or internal structures) within 24 hours after rehydration.

#### Experiment I

In experiment I, we tested the resistance of anhydrobiotic individuals of *H*. *dujardini* to the absence of the geomagnetic field. The specimens from the test group were dehydrated and placed in the CIMF. After 21 days, the specimens were removed from the chamber and rehydrated. Concurrently, the dehydrated specimens from the control group spent 21 days in the Control Box. After this period, all specimens were rehydrated for 24 hours in tap water, and living and dead specimens from both groups were counted (S1 Table).

#### Experiment II

In experiment II, we tested the influence of the absence of the geomagnetic field on the process of entering anhydrobiosis. Specimens were dehydrated inside the CIMF, and then the Petri dishes with tardigrades were removed from the chamber. The specimens spent 21 days in this state under the influence of the geomagnetic field in the box, with the same conditions as the control group. The specimens from the control group dehydrated in geomagnetic field conditions, and then spent 21 days in the Control Box. After that period, all specimens were rehydrated for 24 hours in tap water, and living and dead specimens from both groups were counted (S1 Table).

#### Experiment III

In experiment III, we tested the influence of the absence of the geomagnetic field on the process of the returning of anhydrobiotic *H*. *dujardini* to the active state. Specimens were dehydrated outside the CIMF and spent 21 days in the anhydrobiotic state, but under the influence of the geomagnetic field. Subsequently, Petri dishes with *H*. *dujardini* specimens were transferred to the CIMF, and specimens were rehydrated. After dehydration, specimens from the control group spent 21 days in the Control Box, and then were rehydrated in the same conditions. After this period, all specimens were rehydrated for 24 hours in tap water, and living and dead specimens from both groups were counted (S1 Table).

### Statistics

In all of the experiments, we estimated the mortality of *H*. *dujardini* (expressed as the number of dead individuals) in each Petri dish (S1 Table). To avoid pseudo-replication, we calculated the mean mortality (n = 30 individuals) on each Petri dish; to test differences in mortality between both groups we used a one-tailed t-test, whereas to test differences between the experiments, we used a two-tailed ANOVA [54]. All calculations were performed with the use of R 3.2.3 [55].

## Results

### Experiment I–testing the resistance of anhydrobiotic individuals to hypomagnetic conditions

The mean (±SD) mortality in the test group was 80.3% (±7.7%), and ranged from 70% (21 individuals) to 93.3% (28 individuals), while in the control group the mean mortality amounted to 77.7% (±9.55%), and ranged from 60% to 90% (Table 1). The differences between both groups were not significant (t_18_ = 0.65, p = 0.5).

**Table 1:** Results of Experiment I. Mortality of anhydrobiotic specimens of *H*. *dujardini* (ID- number of Petri dish, LS-live specimens, DS-dead specimens, M-mortality).

ID	Test Group			Control Group		
	LS	DS	M	LS	DS	M
**1**	6	24	80%	12	18	60%
**2**	5	25	83.3%	6	24	80%
**3**	9	21	70%	4	26	86.7%
**4**	4	26	86.7%	5	25	83.3%
**5**	2	28	93.3%	10	20	66.7%
**6**	6	24	80%	6	24	80%
**7**	7	23	76.7%	10	20	66.7%
**8**	3	27	90%	7	23	76.7%
**9**	9	21	70%	3	27	90%
**10**	8	22	73.3%	4	26	86.%
**Total**	**59**	**241**	**-**	**67**	**233**	**-**
**Mean** **(SD)**	**5.9** **(2.3)**	**24.1** **(2.3)**	**80.3%** **(7.7%)**	**6.7** **(2.9)**	**23.3** **(2.9)**	**77.7%** **(9.55%)**

### Experiment II–testing the influence of hypomagnetic conditions upon entering anhydrobiosis

**Fig 2:**
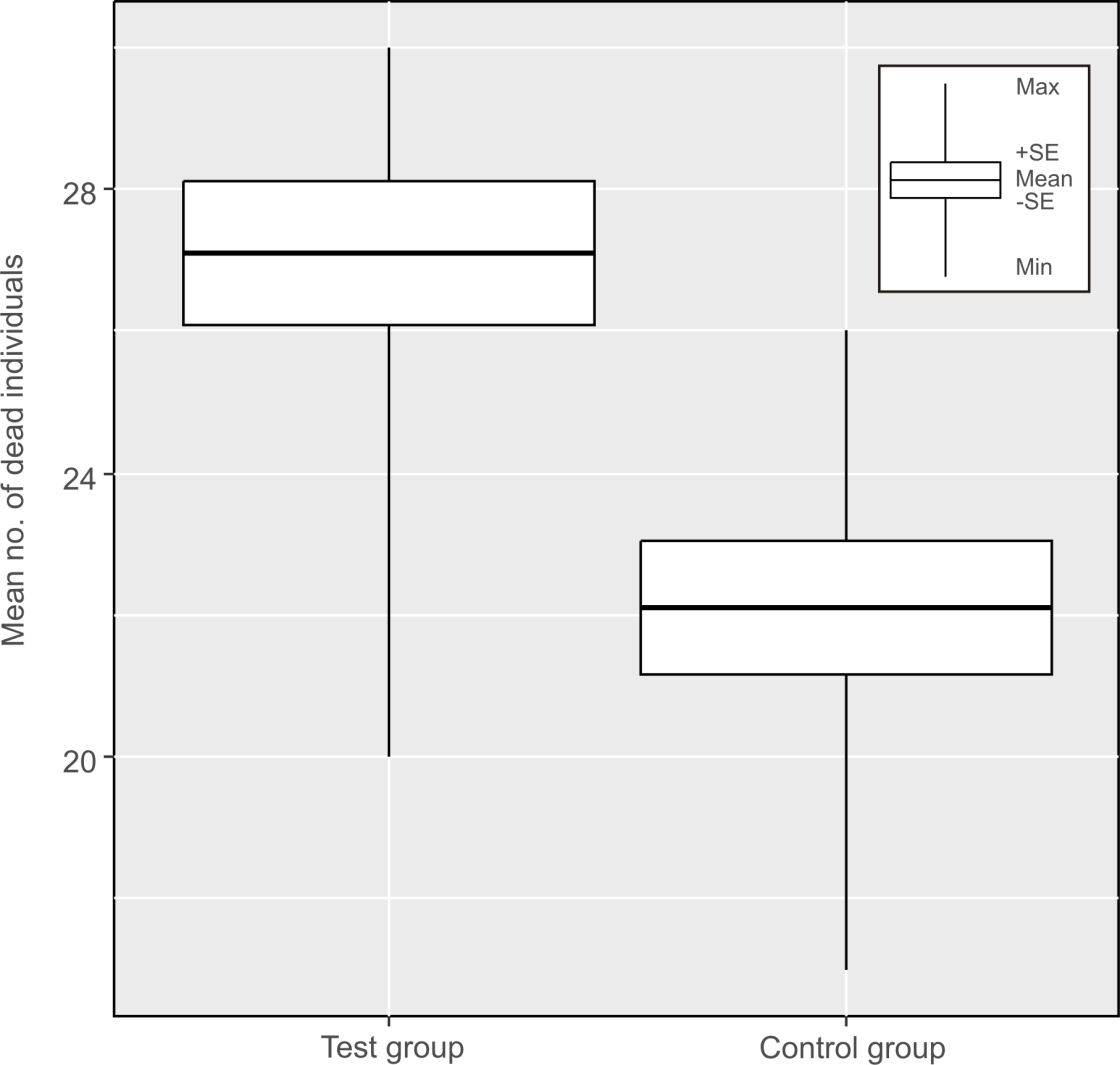
Results of *Experiment II*. Mortality of *H*. *dujardini* during entering into cryptobiosis in isolating chamber (test group) and under influence of geomagnetic field (control group).

In this experiment, tardigrades entering anhydrobiosis were exposed to hypomagnetic conditions, and the mean (±SD) mortality in the test group was 90.3% (±10.2%), ranging from 66.7% (20 individuals) to 100% (30 individuals). Additionally, in 7 of 10 replications, mortality was higher than 90% (Table 2). In the control group, the mean (±SD) mortality was only 73.7% (±9.24), and ranged from 56.7% to 86.7%. Additionally, in 6 of 10 replications, mortality amounted to less than 75% (Table 2). The differences between both groups were statistically significant (t_18_ = 3.6, p = 0.002) (Fig 2).

**Table 2:** Results of Experiment II. Mortality of the *H*. *dujardini* specimens entering anhydrobiosis (ID- number of Petri dish, LS-live specimens, DS-dead specimens, M-mortality).

ID	Test Group			Control Group		
	LS	DS	M	LS	DS	M
**1**	7	23	76.7%	10	20	66.7%
**2**	3	27	90%	8	22	73.3%
**3**	2	28	93.3%	11	19	63.3%
**4**	1	29	96.7%	5	25	83. 3%
**5**	1	29	96.7%	6	24	80%
**6**	3	27	90%	13	17	56.7%
**7**	0	30	100%	9	21	70%
**8**	2	28	93.3%	5	25	83.3%
**9**	0	30	100%	8	22	73.3%
**10**	10	20	66.7%	4	26	86.7%
**Total**	**29**	**271**	**-**	**79**	**221**	**-**
**Mean** **(SD)**	**2.9** **(3.05)**	**27.1** **(3.05)**	**90.3%** **(10.2%)**	**7.9** **(2.7)**	**22.1** **(2.7)**	**73.7%** **(9.2)**

#### Experiment III–testing the influence of hypomagnetic conditions on the process of the return to active life of the anhydrobiotic *H*. *dujardini*

**Fig 3:**
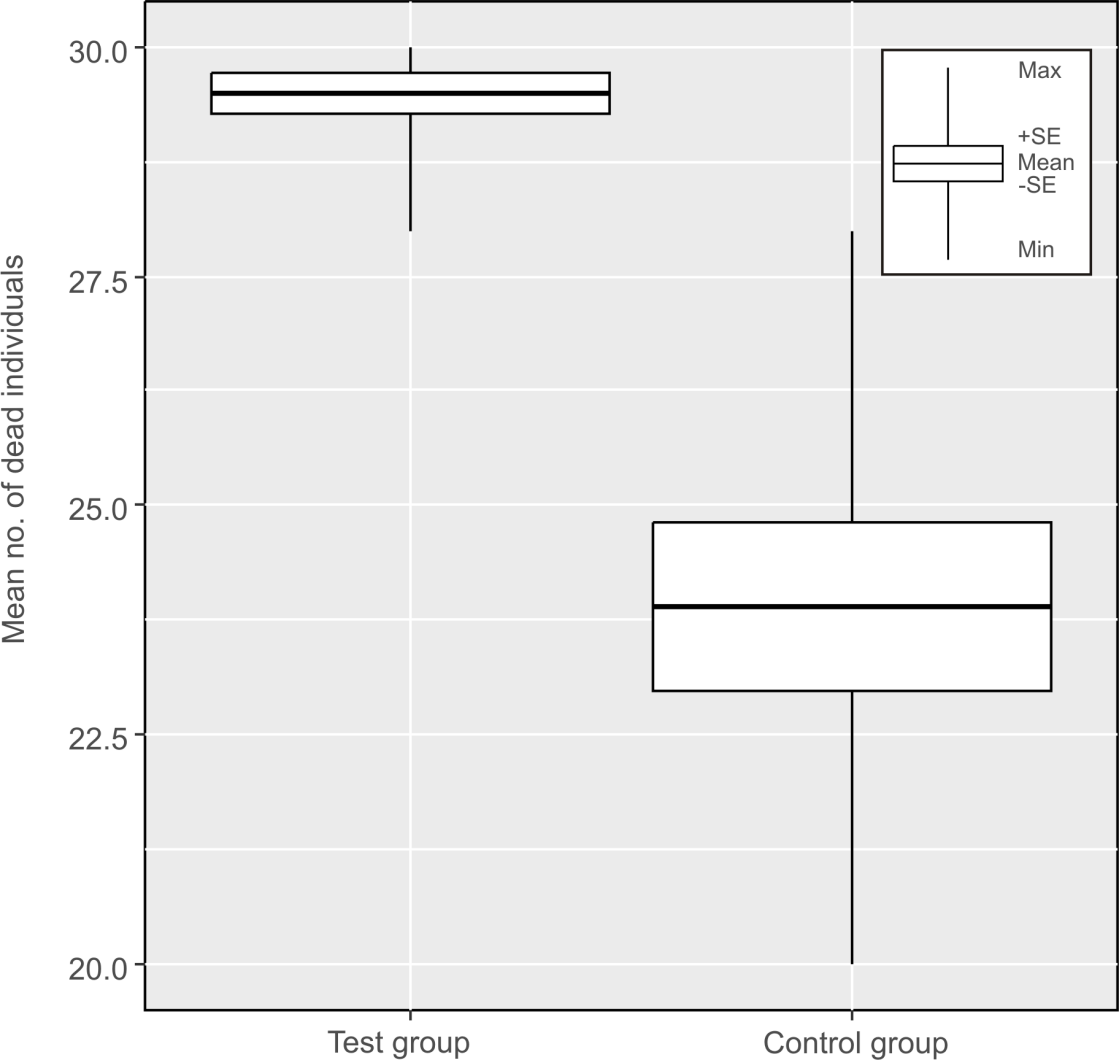
Comparison of the mortality of *H*. *dujardini* returning to active live: In isolating chamber (test group) and under influence of geomagnetic field (control group).

Only five specimens survived in the test group, while more than sixty specimens survived in the control group. The mean (±SD) mortality in the test group was 98.3% (±2.2%) (Table 3), while in the control group the mean (±SD) mortality was only 79.7% (±9.1%) (Table 3, Fig 3). More importantly, mortality in the test group reached 100% in more than half of the replications, while in the control group mortality was higher than 80% only in five replications. Differences in the mean mortality rate between the control and test groups were statistically significant (t_18_ = 5.96, p = 0.00001).

**Table 3:** Results of Experiment III. Mortality of *H*. *dujardini* returning from anhydrobiosis to active life. (ID—number of Petri dish, LS-live specimens, DS-dead specimens, M-mortality).

ID	Test Group			Control Group		
	LS	DS	M	LS	DS	M
**1**	0	30	100%	8	22	73.3%
**2**	1	29	96.7%	3	27	90%
**3**	0	30	100%	5	25	83.3%
**4**	2	28	93.3%	4	26	86.7%
**5**	0	30	100%	10	20	66.7%
**6**	0	30	100%	4	26	86.7%
**7**	1	29	96.7%	2	28	93.3%
**8**	0	30	100%	10	20	66.7%
**9**	0	30	100%	7	23	76.7%
**10**	1	29	96.7%	8	22	73.3%
**Total**	**5**	**295**	**-**	**61**	**239**	**-**
**Mean** **(SD)**	**0.5** **(0.7)**	**29.5** **(0.7)**	**98.3%** **(2.2%)**	**6.1** **(2.7)**	**23.9** **(2.7)**	**79.67%** **(9.1%)**

### Comparison of experiments. Association between *H*. *dujardini* survival rates and the moment (in the life cycle) of isolation from the geomagnetic field

**Fig 4:**
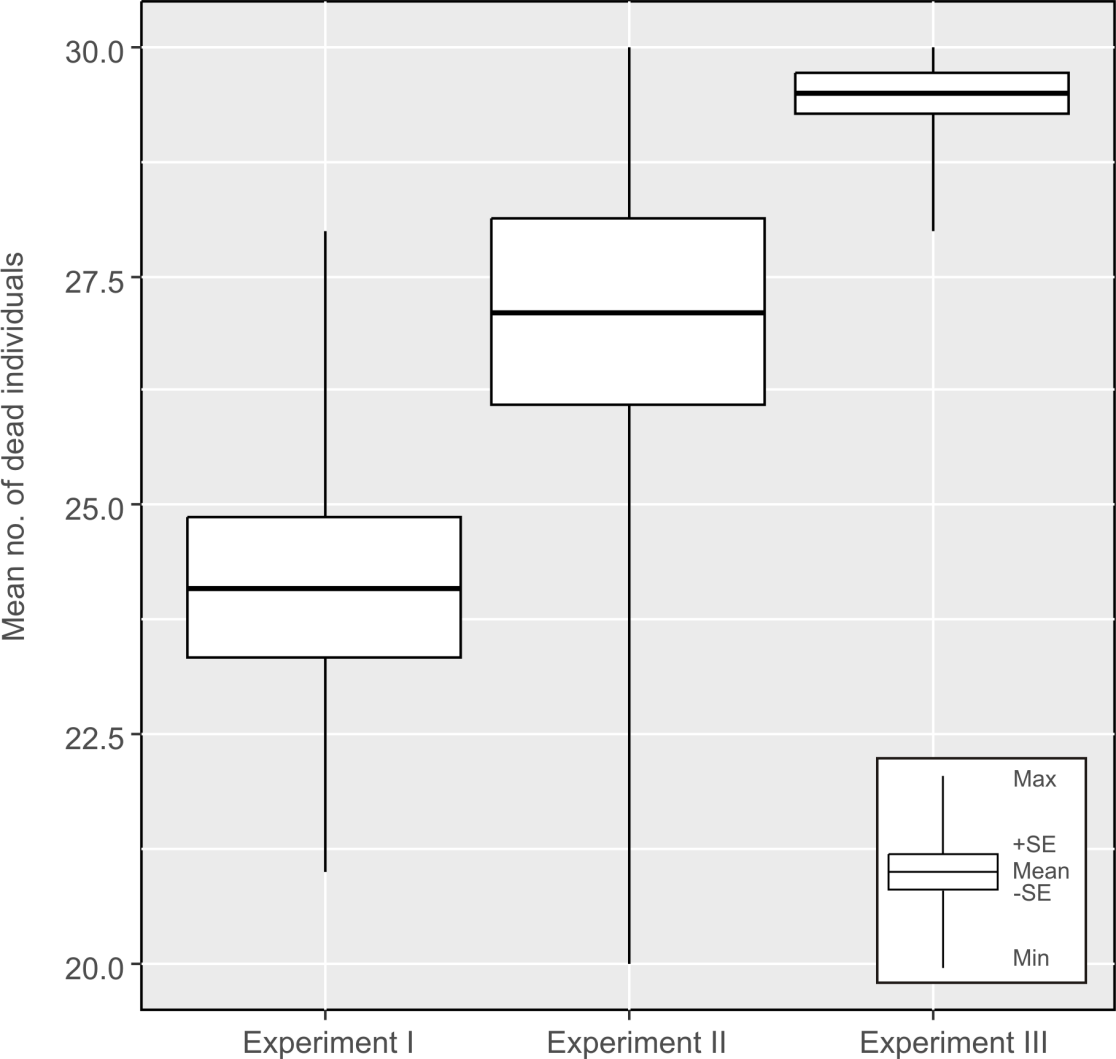
Comparisons of mortalities of *H*. *dujardini* specimens between experiments I, II and III.

We found significant differences between all experiments (ANOVA F_2,27_ = 13.15, p = 0.0001). The largest differences were found between the first *vs*. the second experiment (post-hoc Tukey: p = 0.02), and between the first *vs*. the third experiment (post-hoc Tukey: p = 0.0002). The test group from the first experiment had the lowest mortality, and it was very similar to the mortality observed in the control group. The highest mortality was observed in the test group in the third experiment, however, the average mortality rate in the second and third experiment was not significantly different (post-hoc Tukey: p = 0.77) (Fig 4).

We did not find significant differences in mortality between control groups in different experiments (ANOVA: F_2,27_ = 0.97, p = 0.4).

## Discussion

Our experiments demonstrated that the two critical points for *H*. *dujardini* (and probably also for all other tardigrades capable of anhydrobiosis) are entering anhydrobiosis (experiment II) and returning to the active state (experiment III). In both cases, hypomagnetic conditions significantly increased mortality in the test groups. We had expected such results because we were aware that metabolic processes in tardigrades were very intensive while preparing for anhydrobiosis and then returning to active life. Most tardigrades (including *H*. *dujardini*) prepare for anhydrobiosis by forming a tun [29, 53], reducing their metabolism, synthesising and accumulating different types of bioprotectants [19, 21, 32, 33, 34, 35, 36, 37, 38, 39]. The results suggest that all these processes may be potentially disturbed or impaired by the absence of the geomagnetic field, which–in turn–may lead to an incorrect entry into anhydrobiosis and death, or the inability to return to active life. However, more detailed studies are required to show how the lack of the geomagnetic field influences the entire tardigrade metabolism. Conversely, when returning to the active state, many complex repair processes are activated in tardigrade cells, e.g. DNA repair mechanisms [40, 41], which appear to be even more important than the ones employed when entering anhydrobiosis. These processes are responsible for eliminating all damages accumulated during anhydrobiosis. The results of experiments II and III support our predictions that tardigrades entering anhydrobiosis and returning to active life will be most sensitive to hypomagnetic conditions. However, we had expected that differences in mortality between the test and control groups would be greater. We had also presumed that upon returning to active life, repair processes would be more sensitive to hypomagnetic conditions than those activated on entering anhydrobiosis, because repair mechanisms were the last chance for the organism or cell to avoid permanent damages. If protection mechanisms failed (or were less effective) during exposure to unfavourable conditions (i.e. lack of geomagnetic field), damages within cells and tissue would appear. Nevertheless, as long as repair mechanisms work, some damages can be repaired. When the repair mechanism activity is decreased, the damage resulting from the influence of any other stressors will not be repaired. Consequently, this leads to an increase in the mortality of the tested organisms. Therefore, we expected major differences in the mortality of experimental groups in experiments II and III. Still, differences between experiments II and III were not significant, meaning that both the process of entering and returning from anhydrobiosis are probably equally sensitive. On the basis of the obtained results, we may hypothesise that at least some metabolic processes associated with the entry into anhydrobiosis and return to active life could be disturbed or impaired by hypomagnetic conditions. In our experiments, we also observed relatively high mortality of *H*. *dujardini* of more than 70%, even in the control groups. This was probably due to the fact that *H*. *dujardini* is an aquatic species, and has a relatively low tolerance to dehydration in comparison with terrestrial tardigrades [21]. According to recent publications [21, 46], *H*. *dujardini* is unable to undergo anhydrobiosis, even though a recent study has shown that these species belong to a group of tardigrades that represents the so-called “*slow desiccation type*” of anhydrobiosis [47]. The authors [47] have shown that by using a special slow method of dehydration, specimens of *H*. *dujardini* were able to undergo anhydrobiosis. Independently, we used a similar method of dehydration, but it is possible that this factor could have been responsible for very high mortality in the control groups.

It should also be noted that a few specimens survived in each experiment group. Nonetheless, we suppose that inter-individual differences in molecular or/and metabolic processes (e.g. efficiency of repair mechanisms or synthesis of bioprotectants) in *H*. *dujardini* could be responsible for the results, because all specimens (experimental and control groups) were cultured under the same environmental conditions and the only difference was the influence of hypomagnetic conditions.

Many astrobiologists have considered the possibility of the spread of terrestrial organisms to other celestial bodies of the Solar System [56]. Thus, we put forth a more specific question: could Earth organisms survive drifting in space devoid of a constant magnetic field, and successfully colonise other space objects? Such research has never been conducted on invertebrates capable of anhydrobiosis. Yet, at least a few studies have considered the ability of tardigrades to survive when faced with various environmental stressors (also in outer space), but all of these studies have been conducted under the cover of the geomagnetic field [22, 23, 24, 25, 26, 27, 28, 29, 30, 31, 46, 57, 58, 59, 60, 61, 62, 63, 64]. Space experiments on anhydrobiotic tardigrades were carried out within several projects, i.e. a) RoTaRad—Rotifers, Tardigrades, and Radiation (*Richterius coronifer* (Richters, 1903) and *Milnesium tardigradum* Doyère, 1840), b) TARDIS—TARDigrades In Space (*Richterius coronifer* and *Milnesium tardigradum*) [31], c) TARSE—TArdigrade Resistance to Space Effects (*Paramacrobiotus richtersi* (Murray, 1911)) (all three during the FOTON-M3 mission in 2007), and, d) TARDIKISS—Tardigrades in Space (*Paramacrobiotus richtersi* and *Ramazzottius oberhaeuseri* (Doyère, 1840)) (during the Shuttle STS-134 Endeavour mission in 2011). Yet all of them took place in Low Earth Orbit (LEO) [31, 63, 64]. Mean altitudes of the FOTON-M3 mission were approx. 280 km, and 321–343 km in case of the Shuttle STS-134 Endeavour mission. This means that despite extreme environmental conditions, tardigrades are still protected by the geomagnetic field that surrounds the Earth up to a minimum of 64,000 km (sunward). The results of studies conducted on permanently active individual cells, tissue, plants, or even several species of vertebrates (e.g. mice [9] or rats [10]) in hypomagnetic conditions are hardly comparable to our results obtained for cryptobiotic *H*. *dujardini*.

It is obvious that the absence of the geomagnetic field does not influence specimens of *H*. *dujardini* (and probably also all other tardigrades capable of anhydrobiosis) as long as they are in a permanent anhydrobiotic state. This is because tardigrade metabolism in anhydrobiosis is very slow or completely arrested. In such a situation, hypomagnetic conditions cannot influence these organisms (neither positively nor negatively) because–as previous studies confirm–the absence of the magnetic field can only influence metabolically active cells [11, 12, 13, 14, 15, 16] or tissue [5, 9, 10].

In summary, our study shows that the absence of the geomagnetic field has a significant influence on *H*. *dujardini*. After entering anhydrobiosis under the influence of Earth’s magnetic field, this species can survive away from the geomagnetic field (e.g. on planets or moons without a protective magnetic field or in outer space). They are also capable of returning to the active state in optimal conditions, including the presence of a magnetic field similar in character to the geomagnetic field. However, their return to the active state–even if all other conditions, e.g. liquid water, temperature, and presence of oxygen are suitable–in hypomagnetic conditions of other planets or moons (e.g. Mars) would probably have serious negative consequences. It seems that all organisms living on Earth probably bear the stigmas of geomagnetic interactions that are reflected in the functioning of cells, tissue, organs, systems, as well as entire organisms. In order to understand all these mechanisms, as well as positive or negative consequences of hypomagnetic conditions, additional and more comprehensive studies should be undertaken.

## Conclusions

1. Hypomagnetic conditions have no influence on the mortality of *H*. *dujardini* as long as they are in the anhydrobiotic state with low or no metabolism when they are isolated from the geomagnetic field during anhydrobiosis.

2. Hypomagnetic conditions increase the mortality of *H*. *dujardini* when they enter anhydrobiosis and return to active life.

## Supporting information

S1 Table Detailed information about number of death specimen in each petri dishes. (XLSX)
